# Bifidobacterium pseudocatenulatum capsular exopolysaccharide enhances systemic anti-tumour immunity in pre-clinical breast cancer

**DOI:** 10.21203/rs.3.rs-9946512/v1

**Published:** 2026-06-10

**Authors:** Stephen Robinson, Christopher Price, Alicia Nicklin, Magdalena Kujawska, Todor Koev, Ting Liu, Nilda Ilker, Cristina Lumreras-Perales, Nancy Teng, Wesley Fowler, Alastair McKee, Luke Mitchell, Mitchel Rowe, James Taylor, Christopher Benwell, Sally Dreger, Julia Mueller, Ambrish Kumar, Prithvi Singh, Christian Heiss, Parastoo Azadi, Naiara Beraza, Lindsay Hall

**Affiliations:** University of East Anglia; Quadram Institute Bioscience; Quadram Institute Bioscience; University of Birmingham; University of East Anglia; Quadram Institute Bioscience; Quadram Institute Bioscience; Quadram Institute Bioscience; Quadram Institute Bioscience; Quadram Institute; Quadram Institute; Quadram Institute Bioscience; Quadram Institute Bioscience; Quadram Institute; Quadram Institute Bioscience; Quadram Institute; Technical University of Munich; University of Georgia, Athens; Univesrity of Georgia; University of Georgia; University Of Georgia; Quadram Institute; Quadram Institute Bioscience

**Keywords:** Breast cancer, microbiome, Bifidobacteria, exopolysaccharide (EPS)

## Abstract

Gut microbes have emerged as powerful regulators of cancer responses, with *Bifidobacterium* species and strains playing a key role in promoting anti-tumour immunity. While they represent promising candidates for cancer therapeutics, the specific underlying microbial mechanisms driving their efficacy remains poorly understood. In this study, we demonstrate the broad potential of *Bifidobacterium* species to inhibit breast cancer progression across multiple pre-clinical mouse models. We identify a novel strain, *Bifidobacterium pseudocatenulatum* 210, which induces systemic anti-tumour immunity and enhances responses to standard-of-care therapies via its cell surface capsular exopolysaccharide (EPS). 210 EPS remains predominantly gut local after oral administration and promotes dendritic cell activation, including preferential activation of small intestinal cDC1, leading to robust CD8^+^ T cell-mediated anti-tumour activity. Comparative structural analyses further support that EPS function is strain dependent. Our findings position *Bifidobacterium* EPS as a novel class of therapeutic compounds with significant potential for cancer treatment.

## Introduction

One of the key challenges of cancer therapy is inconsistent patient response to standard of care treatments. Host-intrinsic factors, particularly the gut microbiome, are now recognised as significant contributors to resistance mechanisms that mediate these variable therapeutic outcomes ^1, 2^. The gut microbiome’s role in regulating cancer progression has been demonstrated in both mice ^3, 4, 5^ and humans ^6, 7, 8^, where specific bacterial species and strains can both protect against cancer progression and enhance responses to standard of care therapy ^3, 9, 10^. However, while the concept of anti-tumour activity between intestinal bacteria is established, most studies to date focus on immunogenic tumours (e.g., melanoma, lung cancer) ^11, 12, 13, 14^ and gastrointestinal cancers like colorectal cancer ^9, 15^. The potential influence of gut bacteria on extra-intestinal non-immunogenic indications, like breast cancer, remains poorly characterised.

Beneficial bacteria, such as *Bifidobacterium* and *Lactobacillus*, are known to inhibit solid tumour types in pre-clinical models ^9, 16, 17^. Several species of *Bifidobacterium*, including *Bifidobacterium bifidum* ^15^, *Bifidobacterium breve* ^18, 19^, and *Bifidobacterium pseudolongum* ^10^ have been linked with anti-tumour immunity. Human studies further support these findings, showing positive correlations between abundances of various *Bifidobacterium* species and improved patient outcomes across multiple cancer types ^11, 14^. Collectively, this growing body of evidence supports *Bifidobacterium*-based therapeutic interventions to treat cancer.

Despite these advances, microbiome-based cancer therapeutics have often lacked a clear mechanistic understanding of the microbial-derived active compounds responsible for their beneficial effects. While, microbial metabolites have been most described ^9, 10, 20, 21^, emerging research highlights the role of microbial structural compounds, such as peptidoglycan and exopolysaccharides (EPS), in modulating protective responses ^15, 22^. These exopolysaccharides, composed of polymeric sugar chains with diverse glycosyl content and structure, can drive specific host immune pathways ^23, 24^. A seminal study by *Vétizou et al.*, ^3^ demonstrated *Bacteroides fragilis* polysaccharide A (PSA) mediates anti-tumour immunity response when combined with immune checkpoint inhibitors. More recently, *Sharma et al.*, ^22^ showed that a strain of *Lactobacillus plantarum* surface polysaccharide promotes anti-tumour immunity through enhanced activity of macrophage iron sequestration pathways, skewing tumour-associated macrophages to an anti-tumour CD8^+^-permissive state. A recent study also reported that EPS preparations from *B. pseudolongum* and *B. pseudocatenulatum* can enhance anti-tumour immunity in colorectal systems ^25^, although the chemical structures which define function and how those structures vary across strains have not been characterised.

In this study, we demonstrate that several species of *Bifidobacterium* (*Bifidobacterium bifidum, Bifidobacterium choerinum*, and *Bifidobacterium pseudocatenulatum*) exhibit therapeutic efficacy in pre-clinical breast cancer models. We show that administration of *B. pseudocatenulatum* 210 reduces primary tumour burden across models of the major breast cancer subtypes and enhances responses to standard-of-care chemotherapy and immunotherapy. We demonstrate a novel mechanism whereby *B. pseudocatenulatum* capsular EPS mediates systemic anti-tumour immunity from the gut in a strain dependent manner. 210-derived EPS promotes dendritic cell activation, including preferential activation of small intestinal cDC1, and drives downstream CD8^+^ T cell-dependent anti-tumour immunity associated with increased tumoural IFNγ^+^TNFα^+^ polyfunctional CD8^+^ T cells. Finally, we show that 210-derived EPS can be isolated and used therapeutically independently of live parental bacteria, positioning *Bifidobacterium* EPS as a promising new class of cancer immunotherapy compounds.

## Results

### A Bifidobacterium cocktail treatment reduces tumour burden in breast cancer models

**Figure 1.**
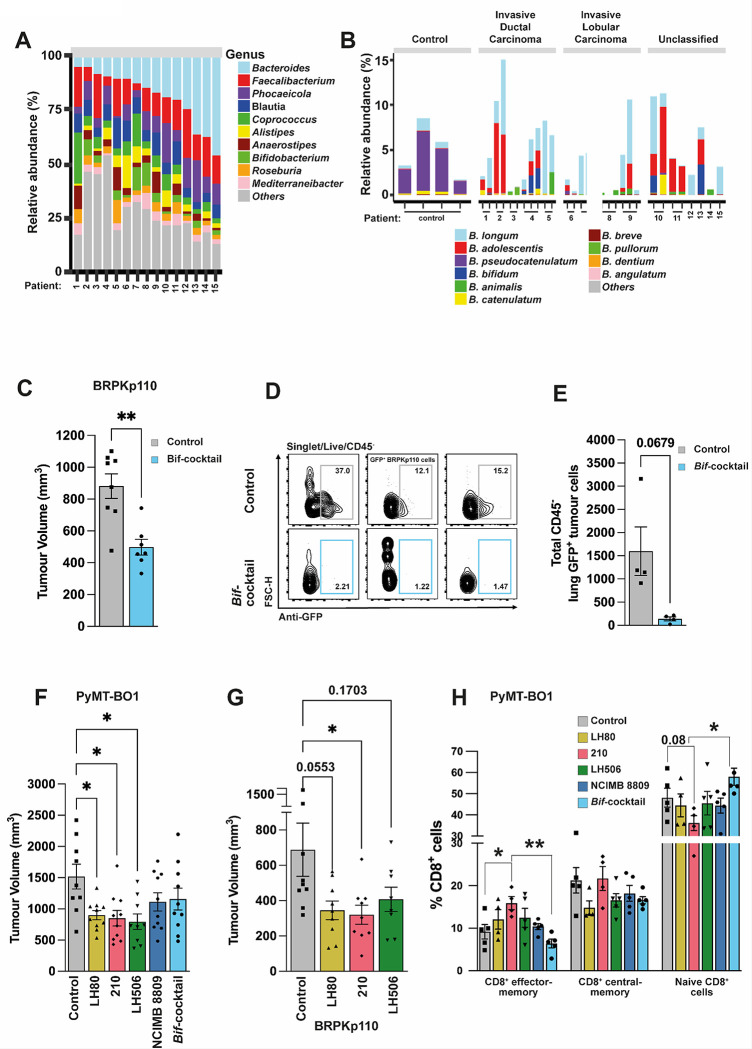
Bifidobacterium is abundant in patients and can induce anti-tumour efficacy in mouse breast models. (A) Relative abundance of the top 10 bacterial genera in faecal microbiomes of breast cancer patients from the BEAM cohort. Samples represent pooled abundances from baseline and post-treatment timepoints. n = 15. (B) Species-level composition of the *Bifidobacterium* genus within the same cohort. Only species with > 1% relative abundance in at least one sample are shown. (C) Endpoint BRPKp110 tumour volumes following oral administration of the Bif-cocktail or PBS vehicle control. n = 7–8. (D) Representative flow cytometry plots showing GFP+ BRPKp110 tumour cells in lungs from control- and Bif-cocktail-treated animals.(E) Quantification of lung GFP+ BRPKp110 tumour cells following Bif-cocktail treatment. n = 4. (F) Endpoint PyMT-BO1 tumour volumes following administration of individual *Bifidobacterium* strains or the 4-strain Bif-cocktail. n = 9–10. (G) Endpoint BRPKp110 tumour volumes following administration of individual *Bifidobacterium* strains. n = 7–9. (H) Quantification of CD8 + T cell differentiation states in PyMT-BO1 tumours following administration of the indicated *Bifidobacterium* treatments. n = 4–5.Statistical significance was assessed by two-tailed unpaired t test in (C,E) and one-way ANOVA with Tukey’s multiple-comparisons test in (F–H). **P < 0.01, *P < 0.05.

To explore the potential relevance of *Bifidobacterium* in breast cancer, we first conducted shotgun metagenomics of faecal material from an initial scoping cohort of breast cancer patients to assess the prevalence and diversity of *Bifidobacterium* species. Analysis of pooled pre- and post-tumour resection samples from 15 patients showed that *Bifidobacterium* was consistently among the top 10 most abundant genera in this cohort (Fig. 1A). Characterisation of the *Bifidobacterium* populations demonstrated a relatively high level of species diversity, with *Bifidobacterium longum, Bifidobacterium bifidum* and *Bifidobacterium adolescentis* among the most highly represented (Fig. 1B). Notably, *Bifidobacterium pseudocatenulatum*, a species typically found in adults, was largely absent from the breast cancer cohort but was detected in healthy control samples. Because this cohort analysis was intended to provide ecological context for *Bifidobacterium* representation rather than define a responder signature, pre- and post-intervention samples were pooled to capture broad species prevalence across the cohort.

Given the observation of a wide diversity of *Bifidobacterium* species prevalent in human patients, as well as an absence of *B. pseudocatenulatum* in the disease cohort, we devised a *Bifidobacterium* cocktail (*Bif*-cocktail) of phylogenetically distinct strains to test potential species-specific effects in pre-clinical tumour models. The *Bif*-cocktail comprised of: *B. longum subsp. longum* NCIMB 8809, *B. bifidum* LH80, *B. pseudocatenulatum* 210, and *B. choerinum* LH506. Oral administration of the *Bif*-cocktail to animals bearing BRPKp110 luminal A-like breast tumours ^26, 27^ led to a significant reduction in BRPKp110 tumour volume (Fig. 1C) and reduced early dissemination of GFP^+^ tumour cells to the lungs (Fig. 1D–E). To dissect the microbial mechanisms driving tumour response, we deconstructed the *Bif*-cocktail, testing the combined treatment against each constituent strain individually in the luminal B-like PyMT-BO1 breast tumour model ^28^. Whilst the *Bif*-cocktail was ineffective in this model, three of the constituent strains (LH80, 210, and LH506) induced an anti-tumour response (Fig. 1F). Comparable tumour reductions were observed after administration of the effective single strains in the BRPKp110 model (Fig. 1G), albeit with slightly less potent efficacy with strain LH506. The activity of CD8^+^ T cells is consistently associated with the anti-tumour activity of *Bifidobacterium* ^4, 10, 29^ and is a vital pathway mediating patient outcomes. Only strain 210 treatment induced CD8^+^ polarisation to an effector-memory subtype in PyMT-BO1 tumours (Fig. 1H), suggesting a CD8^+^T cell-dependent mechanism driving 210 efficacy, and alternative mechanisms mediating response to LH80 and LH506.

### B. pseudocatenulatum 210 exhibits consistent anti-tumour activity across breast cancer models and enhances standard-of-care therapy

**Figure 2.**
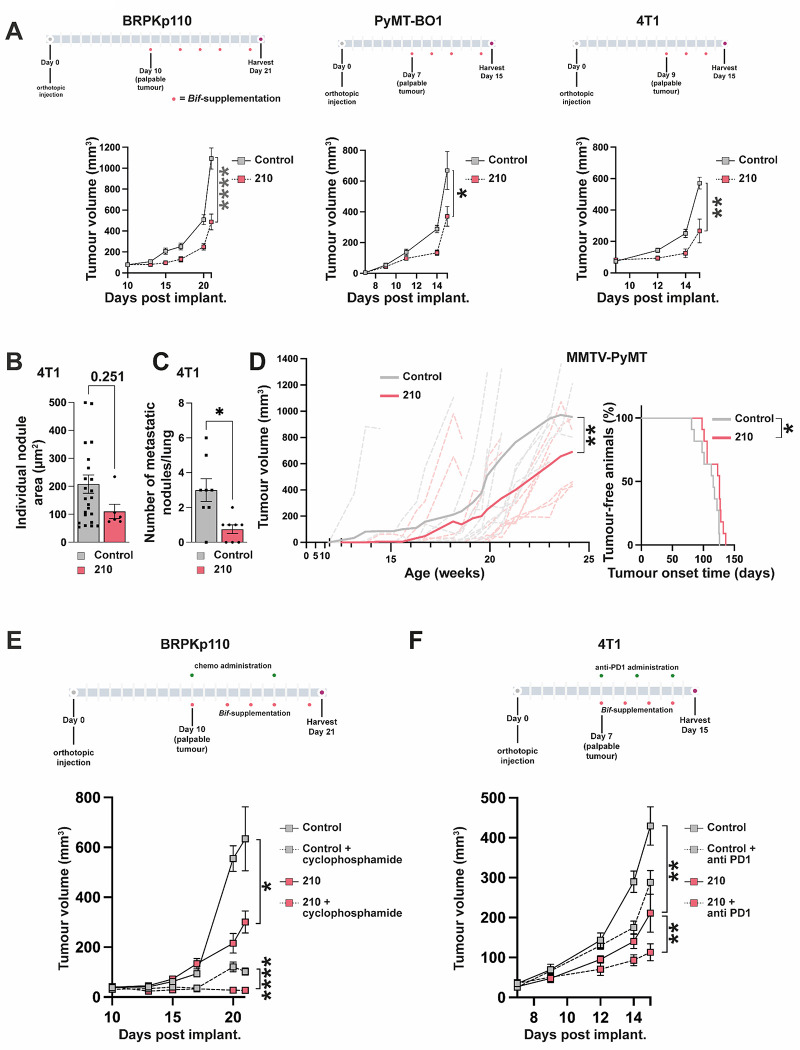
B. pseudocatenulatum 210 inhibits breast tumour progression and enhances responses to standard-of-care therapy. (A) Experimental outlines and tumour growth curves for BRPKp110, PyMT-BO1 and 4T1 orthotopic tumour models following oral administration of *B. pseudocatenulatum* 210 or PBS control. BRPKp110, n = 17–18, N = 2; PyMT-BO1, n = 8–9; 4T1, n = 9. (B) Quantification of individual metastatic nodule area in lungs from 4T1-bearing animals. n = 6–24 nodules. (C) Quantification of metastatic nodules per lung in 4T1-bearing animals. n = 9. (D) Tumour growth and tumour-free survival in the spontaneous MMTV-PyMT model following oral administration of 210. n = 12. (E) Experimental outline and BRPKp110 tumour growth curves following treatment with 210, cyclophosphamide, or the combination. n = 6–8. (F) Experimental outline and 4T1 tumour growth curves following treatment with 210, anti-PD-1, or the combination. Statistical significance was assessed by two-tailed unpaired t test in (A,C,E,F), Mann-Whitney test in (B), and Kaplan-Meier analysis in (D). ****P < 0.0001, **P < 0.01, *P < 0.05.

We focused on *B. pseudocatenulatum* 210 due to its strong and consistent anti-tumour effects. When administered as a monotherapy, 210 induced consistent anti-tumour efficacy across several pre-clinical orthotopic models representing luminal A (BRPKp110), luminal B (PyMT-BO1), and triple negative (4T1) breast cancer – significantly reducing tumour burden (Fig. 2A). In addition, histological analyses showed that 210 reduced 4T1 metastatic burden in the lungs, demonstrating activity beyond the primary tumour site (Fig. 2B–C). In the autochthonous PyMT model, 210 monotherapy not only reduced tumour burden, but also delayed tumour formation, suggesting its potential for longer-term anti-tumour activity and prophylactic utility (Fig. 2D). Furthermore, administration of 210 enhanced the efficacy of standard-of-care treatments, including cyclophosphamide chemotherapy in luminal BRPKp110 tumours and anti-PD-1 immunotherapy in 4T1 (Fig. 2E–F).

### CD8 + T cells drive the anti-tumour response to B. pseudocatenulatum 210

**Figure 3.**
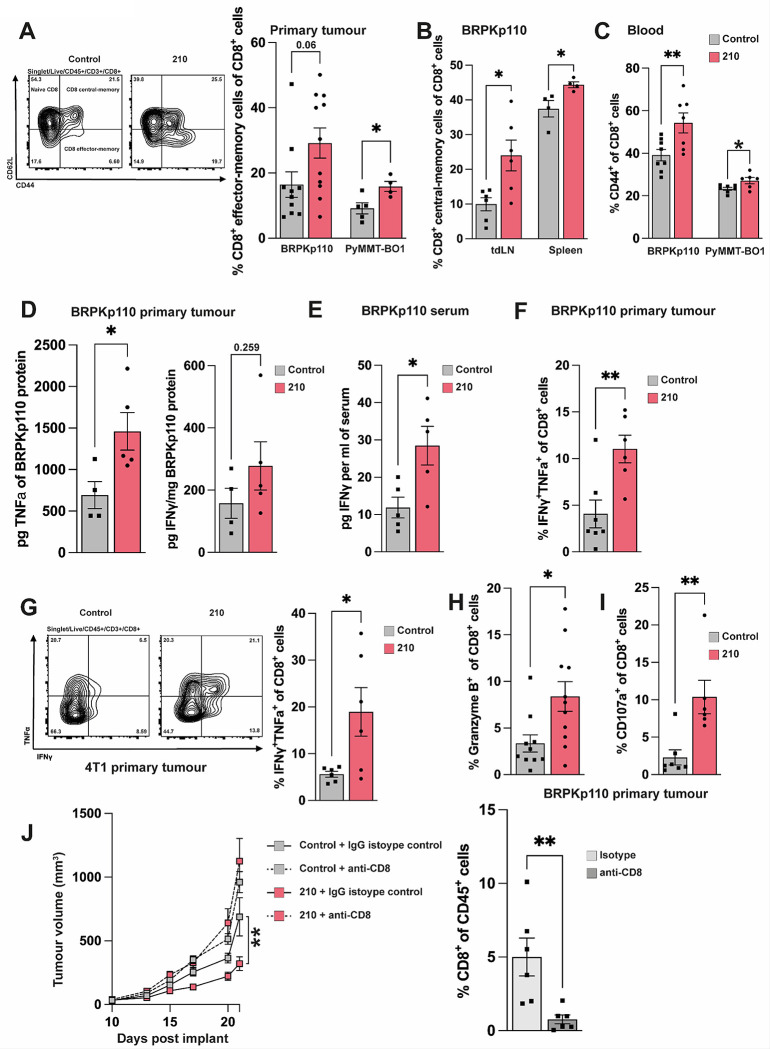
B. pseudocatenulatum 210 anti-tumour efficacy is associated with enhanced CD8 + T cell activation and is CD8 dependent. (A) Representative flow cytometry plots and quantification of CD8 + effector-memory differentiation in primary tumours from BRPKp110 and PyMT-BO1-bearing animals following 210 administration. BRPKp110, n = 11–12; PyMT-BO1, n = 5. (B) Quantification of CD8 + central-memory cells in tumour-draining lymph nodes (tdLN) and spleens of BRPKp110-bearing animals. n = 6 (tdLN), n = 4 (spleen). (C) Quantification of circulating CD44 + CD8+ T cells in blood from BRPKp110- and PyMT-BO1-bearing animals. n = 8 and n = 7, respectively. (D) Quantification of TNFα and IFNγ levels in BRPKp110 primary tumours measured by MSD. n = 4–5. (E) Quantification of serum IFNγ in BRPKp110-bearing animals measured by MSD. n = 5. (F) Quantification of IFNγ + TNFα + CD8 + T cells in BRPKp110 primary tumours. n = 6–7. (G) Representative flow cytometry plots and quantification of IFNγ + TNFα + CD8 + T cells in 4T1 primary tumours. n = 6. (H) Quantification of granzyme B+ CD8 + T cells in BRPKp110 primary tumours. n = 11–12. (I) Quantification of CD107a+ CD8 + T cells in BRPKp110 primary tumours. n = 6–7. (J) BRPKp110 tumour growth curves following treatment with control or 210 in combination with anti-CD8-depleting antibody or isotype control, with accompanying quantification of intratumoural CD8 + T cell depletion. n = 9. Statistical significance was assessed by two-tailed unpaired t test in (A–F,H–J) and Mann-Whitney test in (G). **P < 0.01, *P < 0.05.

Next, we wanted to characterise the immune response driving tumour inhibition of 210. No significant changes were observed in the gross infiltration of major lymphoid populations in the tumour microenvironment (Figure S1A), suggesting immune effects were more likely caused by differential polarisation and activation of immune populations. In agreement with our initial data in PyMT-BO1 tumours, 210 administration also increased BRPKp110 CD8^+^ T cell polarisation towards an effector-memory subtype (Fig. 3A), albeit not to a statistically significant level in the BRPKp110 model. CD8^+^ cells in the spleen and tumour-draining lymph node (tdLN), were more polarised to the central-memory subtype, a state associated with long-lived antigenic memory (Fig. 3B). Additionally, blood circulating CD8^+^ T cells showed a higher frequency of CD44^+^ antigen-experienced cells (Fig. 3C). Cytokine analysis demonstrated that 210 induced increases in TNFα and (non-statistically significantly) IFNy in the primary tumour (Fig. 3D) and were mirrored by a significant increase in serum IFNy levels (Fig. 3E). BRPKp110 tumour infiltrating CD8^+^ cells in both BRPKp110 and 4T1 tumour models (Fig. 3F–G), secreted higher levels of TNFα and IFNy following 210 administration suggesting their involvement in gross increases observed for these cytokines. Enhanced cytolytic activity of CD8^+^ T cells from 210-treated animals was confirmed by increased granzyme B and CD107a expression (Fig. 3H–I). Concurrently, tumour-infiltrating T helper cells were not more polarised or activated, aside from a small increase in IL-4 production restricted to the BRPKp110 model (Figure S1B-E). Likewise, BRPKp110 tumour-infiltrating NK cells were not more abundant and did not produce any higher levels of inflammatory cytokines (including IFNy and TNFα) (Figure S1F), suggesting CD8^+^ T cells may be the sole cytotoxic effector population mediating 210 anti-tumour response. Notably, depletion of CD8^+^ T cells *in vivo* in BRPKp110 tumour-bearing animals abolished the anti-tumour efficacy of 210, validating a CD8^+^-dependent mechanism of action (Fig. 3J).

### B. pseudocatenulatum 210 enhances DC activation

**Figure 4.**
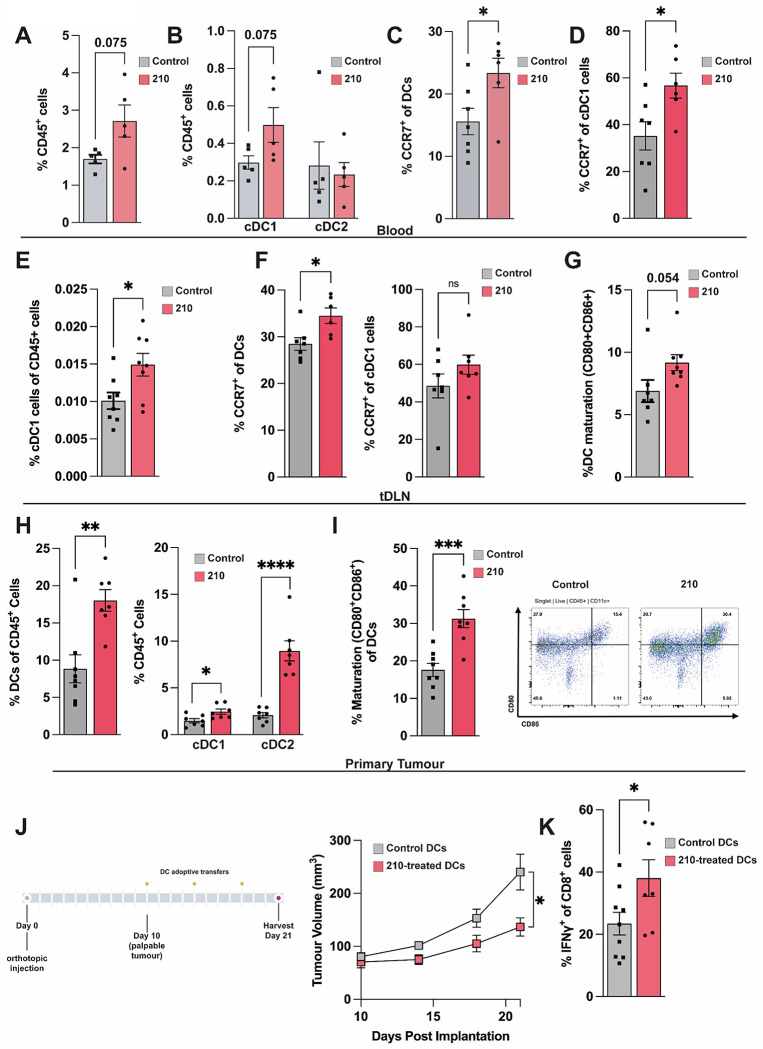
B. pseudocatenulatum 210 enhances dendritic cell activation and primes anti-tumour CD8 + T cell immunity. (A) Quantification of total dendritic cells (DCs) in blood from BRPKp110-bearing animals. n = 5. (B) Quantification of circulating cDC1 and cDC2 subsets in blood. n = 5. (C) Quantification of CCR7 + DCs in blood. n = 6–7. (D) Quantification of CCR7 + cDC1 cells in blood. n = 6–7. (E) Quantification of cDC1 frequency in BRPKp110 tumour-draining lymph nodes (tdLN). n = 8. (F) Quantification of CCR7 + DCs and CCR7 + cDC1 cells in tdLNs. n = 6–7. (G) Quantification of mature (CD80 + CD86+) DCs in tdLNs. n = 7–8. (H) Quantification of total DCs, cDC1 and cDC2 subsets in BRPKp110 primary tumours. n = 7–8. (I) Quantification and representative flow cytometry plots of mature (CD80 + CD86+) DCs in BRPKp110 primary tumours. n = 7–8. (J) Experimental outline and tumour growth curves following adoptive transfer of control or 210-conditioned BMDCs into BRPKp110-bearing animals. n = 8–10. (K) Quantification of intratumoural IFNγ + CD8 + T cells following adoptive transfer of control or 210-conditioned BMDCs. n = 7–9. Statistical significance was assessed by two-tailed unpaired t test, with Welch’s correction applied in (A,B). ***P < 0.001, **P < 0.01, *P < 0.05.

Alongside the CD8^+^ T cell response, we investigated the activity of innate immune cells, given they orchestrate T cell populations. Within BRPKp110 and PyMT-BO1 primary tumours, we observed a significant reduction in CD206^+^ tumour-associated macrophages (TAMs) (Figure S1G). These CD206^+^ TAMs, historically characterised as ‘M2-like’ and pro-tumourigenic ^30^, were replaced with, MHCII+ TAMs (‘M1-like’), indicative of a shift towards a more anti-tumourigenic, CD8^+^-privileged tumour microenvironment (TME). Although the inflammatory state of the TME is vital, we also showed systemic activation of CD8^+^ T cells (in the spleen, tdLN, and blood), which may reflect upstream pathway activation away from the localised TME. Systemic analysis of circulating innate cells highlighted a near-significant increase in the circulating pool of DCs (Fig. 4A).

DC infiltration and activation are pivotal for priming and activation of CD8^+^ T cells due to their role as antigen presenting cells ^31, 32^. Systemic analysis indicated that 210 induced increases in circulating DCs; particularly in the CD8^+^-specific cDC1 population, while no significant changes were observed in the T helper-specific cDC2 population (Fig. 4B). Furthermore, circulating DCs and cDC1 cells primed by 210 displayed enhanced expression of CCR7, consistent with a more activated migratory DC phenotype associated with anti-tumour immune priming ^31^ (Fig. 4C–D). This suggests that 210 may preferentially stimulate CD8^+^ T cell responses via cDC1, pointing to a potential mechanistic cascade for its anti-tumour efficacy. Within the tdLN, a key site for CD8 + T cell programming, we detected an increased frequency of cDC1 cells (Fig. 4E) alongside elevated expression of DC CCR7 (Fig. 4F). Additionally, DCs in the tdLN of 210-treated animals showed elevated expression of CD80^+^CD86^+^ maturation markers, approaching statistical significance (Fig. 4G). Within the primary tumour, we observed a marked increase in the frequency of DCs and cDC1 and cDC2 subsets (Fig. 4H), alongside a concurrent increase in DC maturation (Fig. 4I). Collectively, these data suggest that 210 enhances the systemic activation and maturation-state of DCs, increasing their capacity to support CD8^+^ T cell priming. To further assess whether 210-induced DC activation was directly responsible for the enhanced anti-tumourigenic efficacy and increased CD8^+^ activity, we co-cultured BMDCs with 210 cells and adoptively transferred the treated BMDCs to naïve animals bearing BRPKp110 tumours (Fig. 4J). Notably, 210-conditioned BMDC treatment significantly reduced BRPKp110 tumour burden and enhanced CD8^+^ cell activity within the TME, as evidenced by increased production of inflammatory IFNy cytokine by CD8^+^ cells. This confirms that 210-activated DCs are capable of robustly stimulating anti-tumour CD8^+^ T cell immunity (Fig. 4K).

### B. pseudocatenulatum 210 anti-tumour effects are mediated by EPS

Immunogenic metabolite secretion is a commonly described mechanism in bacterial-mediated cancer immunity ^33^. Untargeted serum metabolomics of 210-treated BRPKp110 and PyMT-BO1 animals did not identify significant changes to the global metabolome profile or individual circulating metabolites and highlighted no consistency across tumour models (Figure S2A-C), suggesting that circulating metabolites were unlikely to underlie the observed anti-tumour effects. In germ-free monocolonised mice, viable 210 colonies were detected only at low levels, mainly in the caecum and colon, and were largely cleared within 24h (Figure S3A). Species-specific (GroEL) qPCR in wild-type animals similarly showed the strongest 210 signal in the caecum/large intestine with decline towards baseline by 48h, although GroEL signal remained detectable in faeces beyond culture positivity (Figure S3B-C), consistent with transient exposure and persistence of non-viable bacterial material. We also found no evidence of direct tumour colonisation by 210 by qPCR or culturomics (Figure S3D-E). Moreover, depletion of the resident microbiota with broad-spectrum antibiotics did not rescue tumour growth in 210-treated animals (Figure S3F), indicating that 210 is mechanistically independent of secondary commensals.

**Figure 5.**
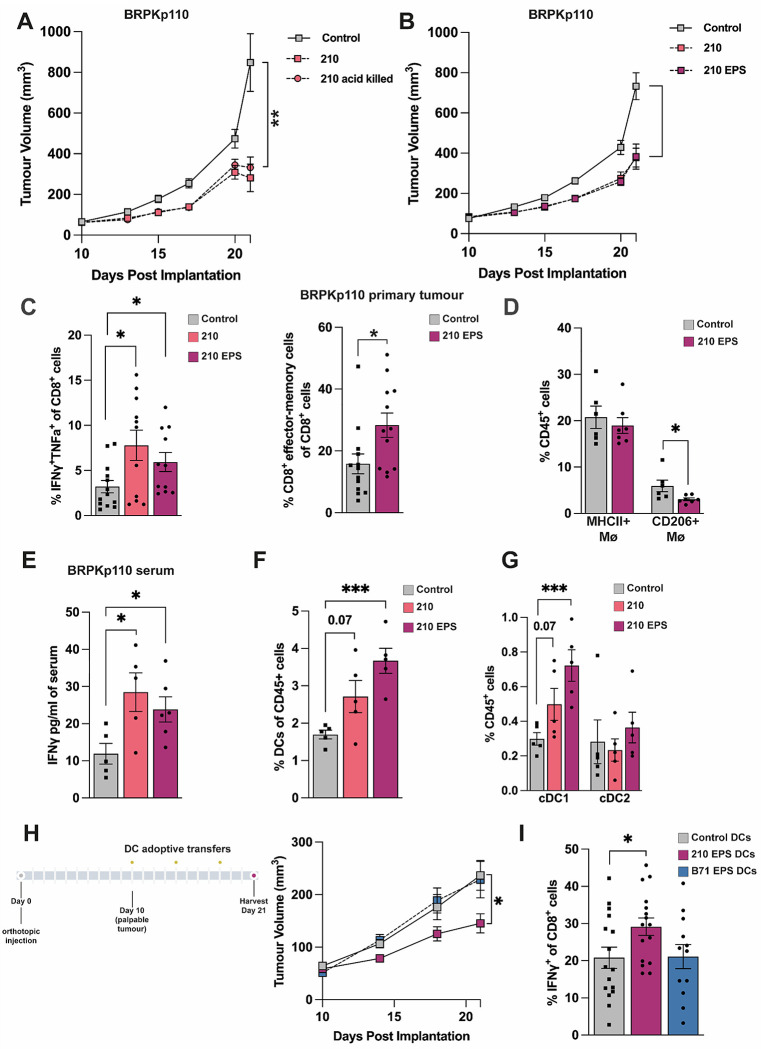
B. pseudocatenulatum 210 anti-tumour activity is mediated by capsular exopolysaccharide. (A) BRPKp110 tumour growth curves following administration of live 210, peracetic acid-killed 210, or PBS control. n = 8–9. (B) BRPKp110 tumour growth curves following administration of live 210, purified 210 EPS, or PBS control. n = 20–23, N = 3. (C) Quantification of IFNγ + TNFα + CD8 + T cells and CD8 + effector-memory differentiation in BRPKp110 primary tumours following treatment with live 210 or purified 210 EPS. n = 11–13. (D) Quantification of MHCII + and CD206 + macrophages in BRPKp110 primary tumours following 210 EPS administration. n = 6–7. (E) Quantification of serum IFNγ in BRPKp110-bearing animals following 210 or 210 EPS treatment. n = 5–6. (F) Quantification of total circulating DCs in blood following treatment with 210 or 210 EPS. n = 5. (G) Quantification of circulating cDC1 and cDC2 subsets in blood following treatment with 210 or 210 EPS. n = 5. (H) Experimental outline and tumour growth curves following adoptive transfer of BMDCs conditioned with vehicle control, 210 EPS or B71 EPS into BRPKp110-bearing animals. n = 18–19, N = 2. (I) Quantification of intratumoural IFNγ + CD8 + T cells following adoptive transfer of control-, 210 EPS- or B71 EPS-conditioned BMDCs. n = 12–16. Statistical significance was assessed by two-tailed unpaired t test. ***P < 0.001, **P < 0.01, *P < 0.05.

These observations suggested a mechanism driven by bioactive structural components rather than active processes, which would rely on viable colonising bacteria. Accordingly, peracetic acid-killed 210 bacterial cells, a method shown by *Moor et al* ^34^ to effectively kill microbes whilst preserving their surface structures, were able to inhibit BRPKp110 tumour growth *in vivo* to a similar extent as live 210 cells (Fig. 5A). Given that *Bifidobacterium* species and strains produce immunogenic EPS which mediate specific immune responses in lymphoid populations ^35^ and DCs ^23^, we tested whether administration of purified 210 EPS (80μg dose delivered orally thrice weekly) was sufficient for activity. Strikingly, 210 EPS treatment mirrored the anti-tumour efficacy (Fig. 5B) and enhanced CD8^+^ T cell cytokine expression and effector-memory differentiation (Fig. 5C) seen with live 210. 210 EPS also reduced the infiltration of CD206^+^ TAMs (Fig. 5D), but did not alter TAM expression of Arg1, iNOS, or PD-L1 (Figure S4A), indicative of a change to lineage infiltrate rather than function. Consistent with live 210, 210 EPS elevated systemic IFNy levels (Fig. 5E), and DC and cDC1 infiltration without altering other lymphoid effector populations, such as T helper or NK cells (Figure S4B-D). Validating that the 210 EPS-specific interactions with DCs were causative, BMDCs conditioned with purified 210 EPS, but not vehicle control or EPS from a non-efficacious control *B. longum* strain (B71), were able to induce significant anti-tumour activity alongside tumour CD8^+^ T cell activation (Fig. 5H–I). CCR7 expression was not enhanced in circulating CD8^+^ T cells (Figure S4E), and CCR7 cognate chemokines CCL19 and CCL21 were not increased in the tdLNs (Figure S4F), suggesting that the 210 EPS mechanism was not driven by an increased chemokine gradient. These data demonstrate that *B. pseudocatenulatum* 210 EPS is the key bioactive compound driving the DC-based immune response, leading to CD8^+^ T cell-mediated tumour suppression.

### B. pseudocatenulatum 210 EPS preferentially activates cDC1 populations in the small intestine

**Figure 6.**
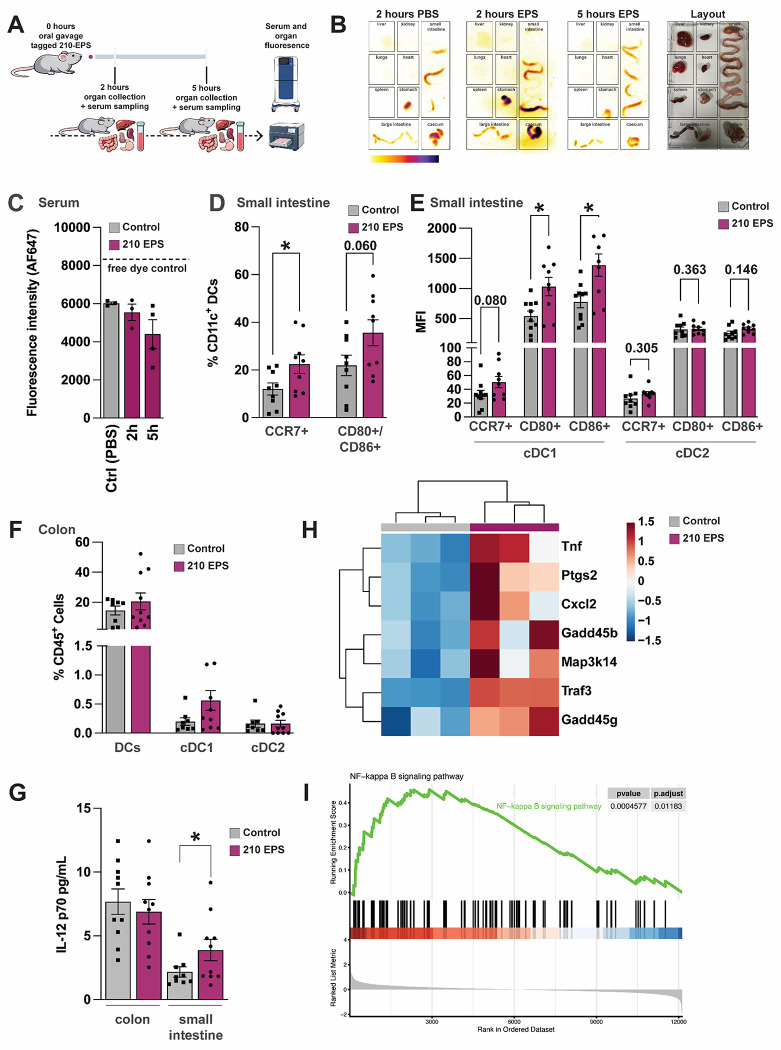
B. pseudocatenulatum 210 EPS remains predominantly gut local and preferentially activates small intestinal cDC1 populations while inducing NF-κB-linked transcriptional reprogramming in BMDCs. (A) Schematic of AF647-labelled 210 EPS oral gavage, serum collection and *ex vivo* organ imaging workflow. (B) Representative *ex vivo* fluorescence imaging of organs collected 2 h after PBS, 2 h after AF647-210 EPS, and 5 h after AF647-210 EPS administration, with organ layout shown at right. (C) Quantification of serum AF647 fluorescence intensity following PBS, 2 h EPS, or 5 h EPS treatment, with free dye control indicated. (D) Quantification of CCR7 + and CD80 + CD86+ total DCs in small intestinal lamina propria following 210 EPS administration. (E) Quantification of CCR7, CD80 and CD86 mean fluorescence intensity in small intestinal cDC1 and cDC2 subsets following 210 EPS administration. (F) Quantification of total DCs, cDC1 and cDC2 frequencies in colonic lamina propria following 210 EPS administration. (G) Quantification of IL-12p70 levels in colon and small intestine following 210 EPS administration. (H) Heatmap showing expression of selected NF-κB-associated genes in BMDCs treated with 210 EPS or PBS control. (I) GSEA plot showing enrichment of the NF-κB signalling pathway in 210 EPS-treated BMDCs. Statistical significance was assessed by two-tailed unpaired t test. *P < 0.05. Raw P values are shown where indicated.

To trace gut-exposed EPS, we orally administered AF647-labelled EPS to tumour-bearing animals (Fig. 6A). *Ex vivo* imaging showed predominant signal in the small intestine and caecum at 2 h, with no detectable organ signal by 5 h (Fig. 6B), and serum AF647 fluorescence was not increased over control (Fig. 6C), suggesting that cell-free soluble AF647-EPS does not translocate systemically in measurable quantities. Since the only detectable location containing 210 AF647-EPS was in the gut, and lamina propria CCR7^+^ DCs can systemically traffic to initiate T cell programs ^36^, we analysed DC infiltration and activation across mucosal tissues. Within the EPS-treated small intestine (SI) lamina propria, DC frequency was significantly higher alongside enhanced CCR7 and CD80/CD86 expression (Fig. 6D), a pattern replicated in the cDC1 but not the cDC2 subset (Fig. 6E). Within the colon, 210 EPS increased cDC1 representation without increasing total DC abundance or maturation markers in bulk DCs, cDC1, or cDC2 (Fig. 6F). Notably, this response was accompanied by increased IL-12p70 in the small intestine, but not the colon (Fig. 6G), further supporting the idea that 210 EPS drives a compartmentalised innate immune programme in the small intestine, where cDC1 accumulation and activation were most evident. Given that simultaneously increased DC and cDC1 infiltration and activation were restricted to the SI, these data are compatible with an SI mucosa-to-lymphoid mechanism that could support the systemic CD8^+^ T cell phenotypes observed.

To better understand how 210 EPS reprograms DCs, we performed RNAseq on 210 EPS–treated BMDCs. EPS exposure induced a transcriptional program consistent with DC activation and enhanced antigen presentation, including significant upregulation of classical and non-classical MHC I genes (H2-K2, H2-Q4, H2-Q5) and inflammatory/activation-associated regulators (e.g., Tnf, Irf1) (Fig. 6H, Figure S5A-B). Notably, Map3k14 (NIK) and Traf3 were significantly increased, supporting engagement of a non-canonical NF-κB regulatory axis. Pathway analysis further highlighted enrichment of NF-κB–linked gene sets and modest induction of mitochondrial electron transport chain transcripts, consistent with broader DC functional reprogramming that could support downstream CD8^+^ T cell immunity (Fig. 6I).

### B. pseudocatenulatum EPS is highly variable and functions in a strain-dependent manner

**Figure 7.**
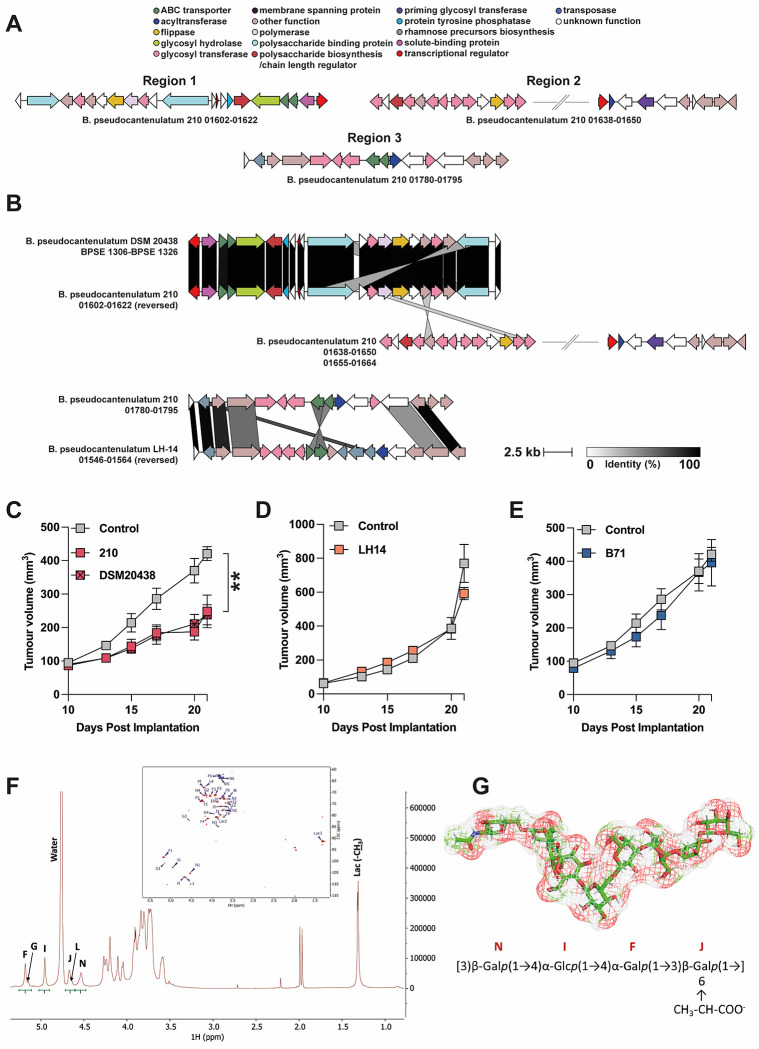
B. pseudocatenulatum EPS function is strain dependent and associated with distinct EPS genomic and structural features. (A) Predicted architecture of putative EPS biosynthetic loci in *B. pseudocatenulatum* 210. (B) Homology maps comparing putative 210 EPS loci with those of *B. pseudocatenulatum* DSM20438 and LH14. (C) BRPKp110 tumour growth curves following administration of *B. pseudocatenulatum* 210 or DSM20438. n = 8–9. (D) BRPKp110 tumour growth curves following administration of *B. pseudocatenulatum* LH14. n = 6–7. (E) BRPKp110 tumour growth curves following administration of *B. longum* B71. n = 8. (F) ^1^H NMR spectrum and (inset) 2D NMR data for 210 EPS fraction 1. (G) Structural model of the major lactate-modified repeating unit identified in 210 EPS fraction 1: [3)-β-Galp-(1→4)-α-Glcp-(1→4)-α-Galp-(1→3)-β-Galp-(1→]. Statistical significance was assessed by two-tailed unpaired t test in (C–E). **P < 0.01.

To gain insight into the uniqueness of 210 EPS relative to other strains and species of *Bifidobacterium*, we undertook comparative analyses of the putative EPS enzymatic clusters within the genomes of the strains used in the study. Predictive EPS biosynthetic loci showed marked diversity across *Bifidobacterium* species, with *B. pseudocatenulatum* 210 EPS being highly distinct from *B. bifidum* LH80, *B. choerinum* LH506, *B. longum subsp. longum NCIMB* 8809, and *B. longum* B71 genomes (Figure S6A-B). Comparison against other strains of B. *pseudocatenulatum* showed high levels of genetic homology between 210 and the *B. pseudocatenulatum* DSM20438 type strain, with a low level of homology *B. pseudocatenulatum* LH14 (Fig. 7A–B). Given the similarity of the putative EPS genes encoded by 210 and DSM20438, and the large differences to those of LH14, we tested whether these differences would be reflected functionally in (EPS-driven) tumour reduction. Indeed, administration of DSM20438 resulted in significant tumour inhibition comparable with that of 210 (Fig. 7C), whilst strains LH14 and B71 were ineffective in reducing tumour progression (Fig. 7D–E). Consistent with this, DSM20438 recapitulated the key immune features of 210, including enhanced tumoural CD8^+^ T cell activation/effector-memory differentiation and increased DC infiltration/maturation, whereas LH14 did not (Figure S7A-G). *In vitro* analysis of BMDCs exposed to purified EPS from 210, DSM20438 and *B. longum* B71 further mirrored the functional differences observed *in vivo*. Although each EPS sample was able to stimulate the BMDC maturation in a dose-dependent manner, EPS from 210 and DSM20438 demonstrated greater BMDC stimulatory MHCII expression and CD80^+^CD86^+^ maturation at a lower dose (10μg) than the B71 EPS (Figure S7H).

To understand the structure-function relationship mediating the biological activity of 210 and B71 EPS, we undertook structural characterisation by glycosyl composition and linkage analysis, size exclusion chromatography (SEC) and NMR spectroscopy. Purified 210 EPS comprised a heterogeneous galactose- and glucose-rich polysaccharide preparation that resolved into three fractions by SEC, whilst the more glucose-dominant B71 EPS resolved to four fractions (Figure S8A). To identify the 210 EPS fraction most likely to contain DC-interacting material, we performed on-cell saturation transfer difference (STD) NMR using BMDCs. Fraction 1 showed *circa* 10-fold higher binding affinity versus fractions 2 and 3 (Figure S8B). 2D NMR analysis of 210 fraction 1 revealed a unique lactate-modified galactose- and glucose-containing polysaccharide (Fig. 7F). ^1^H-^13^C HMBC and ^1^H-^1^H NOESY correlations established a repeating tetrasaccharide unit, 3-β-Galp(1→4)α-Glcp(1→4)α-Galp(1→3)β-Galp-6-lactate (Fig. 7G), in which residues N and J were assigned as 3-substituted β-galactopyranose units, and residues I and F as 4-substituted α-glucopyranose and 4-substituted α-galactopyranose. Additional minor residues were detected but could not be fully resolved (Supplementary Tables 2A/2B). Contrastingly, B71 peak 1 comprised two different major polysaccharide species and B71 peak 2 contained four distinct repeating units, with higher proportions of 2- and 3-linked Glcp residues and more branched 4,6-Galp/5,6-Galf-type linkages. The structures that could be resolved for each fraction of 210 EPS and B71 EPS are shown in Figures S8D-F, with ^1^H and ^13^C NMR chemical shifts of sugar residues identified shown in Supplementary Tables 5–7. Taken together, these data show that the lactate containing high molecular weight fraction 1 in the 210 EPS is highly unique from the ineffective B71 EPS, and is key in mediating biological interactions with DCs and thus stimulating downstream anti-tumour immunity.

## Discussion

Therapeutically harnessing gut microbes to treat chronic disease is a fundamental aim in microbiome research. Whilst numerous studies have highlighted the potential of microbiota-based interventions, translation to extra-intestinal disease therapies remains limited. A significant barrier is our incomplete understanding of the specific microbial-derived functional compounds that drive therapeutic responses. Without identifying these key compounds, we cannot control how they may be produced or influenced in patients. In this study, we demonstrate the broad potential of various species of *Bifidobacterium* to treat breast cancer. We provide strong evidence that *B. pseudocatenulatum* 210 inhibits breast tumour progression and enhances the efficacy of standard-of-care therapies through presentation of cell surface capsular EPS. Together with a recent report showing that bifidobacterial EPS can promote anti-tumour immunity in colorectal models ^25^, our data strengthen the case for *Bifidobacterium* EPS as an anti-tumour modality. Importantly, we identify a strain-resolved, structurally characterised capsular EPS from *B. pseudocatenulatum* 210 that acts in breast cancer models and links gut-local exposure to systemic anti-tumour immunity.

The link between *Bifidobacterium* and cancer has been an active area of research for the past decade. *Sivan et al* ^4^ were the first to show that a cocktail of *Bifidobacterium* species enhanced immune checkpoint inhibitor (ICI) responsiveness in melanoma through a DC and live bacteria-dependent mechanism. Our findings further support the anti-tumour potential of single *Bifidobacterium* strains ^10, 15, 19^, while also highlighting substantial species- and strain-level functional heterogeneity within the genus, meaning that genus-level abundance may obscure the relative representation of beneficial versus non-beneficial taxa; notably, *B. pseudocatenulatum* itself was largely absent from our disease cohort despite its potent activity in our pre-clinical models. The human cohort component of this study was therefore intended to provide ecological context for strain selection rather than to establish clinical association or predictive value, which will require larger, prospectively designed patient studies.

In this study, we observed that a Bifidobacterium (Bif)-cocktail induced anti-tumour efficacy in the luminal A (BRPKp110) but not luminal B (PyMT-BO1) model. Three of the four component strains (*B. bifidum* LH80, *B. choerinum* LH506, and *B. pseudocatenulatum* 210) independently inhibited PyMT-BO1 tumour progression, whereas *B. longum* NCIMB 8809 did not. However, only *B. pseudocatenulatum* 210 induced CD8 + effector-memory polarisation, whereas LH80 and LH506 did not. Together with the marked diversity in predicted EPS loci across these strains, this suggests that although multiple bifidobacterial strains may have therapeutic potential against breast cancer, the underlying molecular mechanisms are likely distinct. In the present study, we focused mechanistic analysis on 210 and did not obtain equivalent structural resolution of LH80- or LH506-derived EPS or systematically define the alternative immune pathways through which these strains may act. These remain important priorities for future work.

Our investigation into *B. pseudocatenulatum* 210 revealed potent anti-tumour effects across multiple breast cancer models, including long-term efficacy in the spontaneous PyMT model and enhanced responses to chemotherapy and ICI treatment. Mechanistically, these effects converged on a DC–CD8 + T cell axis, with dendritic cell activation and downstream CD8 + T cell reprogramming emerging as the dominant immune features associated with efficacy.

A point of difference to many existing microbiome-cancer studies(cites) was that neither bacterial colonisation nor viability was required for efficacy, and that 210 did not induce significant changes to the metabolome. Rather, acid killed 210 remained efficacious, suggesting structural components mediated response. In our study, isolated EPS from *B. pseudocatenulatum* 210 replicated the tumour inhibition and immune-stimulatory effects of live 210, and adoptive transfer of BMDCs conditioned with 210-derived EPS further confirmed its role as the critical mechanistic driver. The ability of purified 210 EPS to recapitulate the anti-tumour activity of live bacteria has strong translational appeal, as it may circumvent some of the colonisation, engraftment and manufacturing challenges associated with live microbial therapeutics.

Our analyses suggest that the EPS cascade is initiated locally within the gut mucosa. Orally administered AF647-labelled 210 EPS accumulated predominantly in the gut (small intestine and caecum), where it was associated with SI lamina propria cDC1 and increased intestinal IL-12. Given that intestinal lamina propria dendritic cells can acquire mucosal antigen and traffic via lymphatics to mesenteric lymph nodes to support downstream T cell priming ^37^, these data are consistent with a mucosa-to-lymphoid relay linking gut-local EPS sensing to systemic anti-tumour immunity. BMDC RNA-seq further supported direct functional reprogramming by 210 EPS, revealing induction of antigen presentation and NF-κB-linked activation programmes. These findings align in part with recent evidence that bifidobacterial EPS can induce dendritic-cell IL-12/TNF-α programmes ^18, 25^ but substantially extend that work by localising the initiating response to the small-intestinal mucosa and linking it to preferential cDC1 activation by a structurally characterised, strain-specific 210 EPS.

Comparative analysis of the architecture of putative EPS genomic clusters revealed high levels of genomic variability between individual strains and species, but also that genetic homology of EPS-synthesising clusters correlated with function *in vivo* and *in vitro*. This was demonstrated by overlapping responses to *B. pseudocatenulatum* 210 and *B. pseudocatenulatum* DSM 20438, which had highly homologous EPS clusters. This contrasted with the absence of functional responses from genetically distinct *B. pseudocatenulatum* LH14 and *B. longum* B71. Importantly, our glycosyl analyses show that active 210 EPS and ineffective B71 EPS also differ in sugar composition, linkage profile, and polysaccharide complexity. Notably, the higher-molecular-weight fraction of 210 was more structurally distinct from B71 with a lactate-substituted repeating unit, and this fraction was revealed to have the highest binding affinity with BMDCs. Together, these findings support that EPS immunostimulatory function is highly strain dependent and shaped by both biosynthetic gene content and resulting polysaccharide structure. More broadly, they suggest that integrated genomic, structural, and functional analysis of EPS may provide a useful strategy to identify immunomodulatory bacterial polysaccharides with therapeutic potential.

The clinical relevance of these findings is underscored by the growing body of evidence linking *Bifidobacterium* species to favourable cancer outcomes ^12, 38^. Of specific interest, is the correlation of *B. pseudocatenulatum*, alongside other beneficial species like *Roseburia* spp. and *Akkermansia muciniphila*, with enhanced overall response rates and progression-free survival in several cohorts of melanoma patients receiving ICIs ^11^. By demonstrating that *B. pseudocatenulatum* EPS drives CD8 + T cell-mediated anti-tumour immunity in breast cancer, we provide a strong foundation for future clinical translation of *Bifidobacterium*-based interventions.

Taken together, our data identify *B. pseudocatenulatum* 210 EPS as a strain-specific immunomodulatory polysaccharide capable of recapitulating the anti-tumour activity of live bacteria through a DC–CD8^+^ T cell axis. The gut-local biodistribution, preferential activation of small intestinal cDC1, and direct reprogramming of BMDCs support a model in which 210 EPS initiates a mucosal immune relay with systemic anti-tumour consequences. Structural comparison with B71 further suggests that this activity is linked not simply to the presence of EPS, but to specific EPS architecture, including a distinct lactate-modified high-molecular-weight 210 fraction not observed in the same form in B71. These findings strengthen the rationale for developing bacterial EPS as a therapeutic modality.

### Limitations

Our biodistribution analyses indicate that orally administered AF647-labelled 210 EPS remains predominantly gut local, with the strongest signal detected in the small intestine and caecum. However, organ-level fluorescence imaging and serum fluorescence are relatively insensitive readouts of soluble labelled EPS and may underestimate low-level systemic dissemination, particularly if EPS is transported in a cell-associated rather than soluble form. In addition, these experiments were performed using chemically labelled EPS generated by periodate oxidation and hydrazide coupling, and fluorophore conjugation could alter aspects of native EPS behaviour, including retention, uptake, or trafficking. Thus, although our data strongly support gut retention of the bulk EPS pool, they do not fully exclude limited systemic transfer of native or cell-associated material.

Although we identified preferential activation of small intestinal lamina propria cDC1 following 210 EPS exposure, we did not directly trace the migration of EPS-programmed immune cells from the intestine into Peyer’s patches, mesenteric lymph nodes, blood, or tumour-draining lymph nodes. Accordingly, the present study supports a plausible cDC1-centred mucosal mechanism, but the precise trafficking route linking local EPS exposure to distal anti-tumour immunity remains to be established.

## METHODS

### RESOURCE AVAILABILITY

#### Lead contact

Further information and requests for resources and reagents should be directed to and will be fulfilled by the lead contacts, Lindsay J. Hall and Stephen D. Robinson.

#### Materials availability

This study did not generate unique new reagents that are unrestrictedly distributed through a public repository. Bifidobacterium strains, purified exopolysaccharide (EPS) preparations, and associated materials generated for this study are available from the lead contacts upon reasonable request and subject to any relevant material transfer agreements.

### EXPERIMENTAL MODEL AND STUDY PARTICIPANT DETAILS

#### Human cohort

Faecal samples were collected from participants in the Breast hEalth And Microbiota (BEAM) study, conducted at the Norfolk and Norwich University Hospital and James Paget University Hospital, UK. Ethical approval was granted by the University of East Anglia Faculty of Medical Health Sciences Research Ethics Committee (FMH 201819-092HT) and the National Research Ethics Service (HTA license 11208). Patients were eligible if they were older than 30 years, had at least three months without antibiotic exposure, and had no previous primary cancer diagnosis. All participants provided written informed consent and were issued self-sampling faecal kits for home collection following mammogram screening. Samples were returned to the Quadram Institute Bioscience and processed within 24 h. Participants provided up to four samples over the study period: baseline, post-surgery or therapy, six months, and one year post-enrolment. Breast cancer subtype diagnoses were obtained from standard clinical pathology reports. Faecal samples were aliquoted under sterile conditions and stored at − 80°C until DNA extraction.

#### Mice

Animal studies were designed and reported in line with ARRIVE 2.0 recommendations, with each individual animal considered the experimental unit. C57BL/6J and BALB/c mice were purchased and maintained in-house at the Disease Modelling Unit, University of East Anglia, under project licence code PP8873233 and under specific pathogen-free conditions with ad libitum access to food and water and appropriate environmental enrichment. Female mice aged 8–12 weeks were used for orthotopic tumour studies unless otherwise stated. Animals were age-matched within experiments and randomly mixed between cages prior to experimental onset. Germ-free experiments were performed using mice maintained in-house in isolators under germ-free conditions. MMTV-PyMT mice (B6.FVB-Tg(MMTV-PyVT)634Mul/LellJ) were maintained on a congenic C57BL/6 background. All animal procedures were conducted in accordance with the UK Animals (Scientific Procedures) Act 1986 and approved by the UK Home Office, in line with Directive 2010/63/EU on the protection of animals used for scientific purposes.

#### Bacterial strains

All *Bifidobacterium* strains used in this study were from the laboratory collection of Prof. Lindsay J. Hall and are listed in Supplementary Table 1 of the manuscript. The strains included *Bifidobacterium pseudocatenulatum* 210, *B. pseudocatenulatum* DSM20438, *B. longum subsp. longum* B71, *B. bifidum* LH80, and *B. choerinum* LH506. Where indicated, strains previously described by Lawson et al. (2020) and Kujawska et al. (2020) were used for comparative genomic analyses ^39, 40^.

#### Cell lines

Syngeneic murine mammary tumour cell models used in this study were BRPKp110, PyMT-BO1 and 4T1. Cells were cultured at 37ºC in 5% CO_2_ in DMEM high-glucose medium supplemented with 10% faetal bovine serum (FBS). Bone marrow-derived dendritic cells (BMDCs) were generated from flushed murine bone marrow as described below.

### METHOD DETAILS

#### Human metagenomics

Microbial DNA was extracted from 200 mg of faecal material using the FastDNA SPIN Kit for Soil with a modified protocol incorporating mechanical lysis in Lysing Matrix E tubes using a FastPrep-24 bead beater at 6.0 m/s for 3 min. DNA was eluted, stored at - 20°C, and quantified using a Qubit fluorometer with the dsDNA Broad Range Assay Kit. Shotgun metagenomic libraries were prepared in-house using a modified Illumina DNA Prep tagmentation protocol following the CoronaHiT-Illumina approach ^41^. Libraries were size selected by dual-SPRI bead cleanup, assessed on a TapeStation instrument, pooled, and sequenced on an Illumina NextSeq 500 Mid Output Flowcell (300 cycles, 2 × 150 bp) with a final loading concentration of 1.5 pM and 1% PhiX. Raw reads were quality filtered with fastp v0.20.0 ^42^. Human reads were removed by alignment against the human genome using BBMap, and taxonomic profiling was performed using Kraken2 with abundance refinement by Bracken ^43^. Downstream community analyses were performed in R.

#### Tumour models and in vivo interventions

For orthotopic tumour studies, syngeneic breast cancer cells were injected in 50 μL of a 1:1 mixture of PBS and Matrigel into the left inguinal mammary fat pad of age-matched female mice. PyMT-BO1 and 4T1 cells were injected at 1 × 10^5^ cells per mouse, and BRPKp110 cells at 5 × 10^5^ cells per mouse. Tumour growth was monitored three times per week by digital calipers, and tumour volumes were estimated using the formula length × width^2^ × 0.52 ^44^. For spontaneous tumour experiments, MMTV-PyMT mice were palpated regularly, and treatment was initiated at the onset of measurable disease. When tumours became palpable, mice were randomly allocated to treatment groups, and investigators were not blinded to group allocation. Animals were excluded only if tumours ulcerated.

Animals were orally administered three times weekly with live *Bifidobacterium* strains (1 × 10^10^ CFU in 200 μL PBS) or isolated EPS (80 μg in 200 μL PBS) from the onset of palpable tumour formation until experimental endpoint. For chemotherapy studies, cyclophosphamide was administered intraperitoneally at 100 mg/kg on days 10 and 17. For checkpoint therapy experiments, anti-PD-1 monoclonal antibody (clone 29F.1A12) or matched isotype control was administered intraperitoneally at 20 mg/kg on days 7, 10, and 13. CD8 depletion was induced using anti-CD8α antibody (clone 2.43) or matched isotype control, with 400 μg administered 1 day before bacterial treatment followed by 200 μg on days 13, 16, and 19. For microbiome depletion studies, tumour-bearing mice received an oral antibiotic cocktail containing 0.2 mg/mL amphotericin B, 5 mg/mL vancomycin, 10 mg/mL neomycin, and 10 mg/mL metronidazole, with drinking water supplemented with 1 mg/mL ampicillin. For adoptive transfer experiments, 1 × 10^6^ BMDCs were conditioned with live 210 bacteria, 210 EPS, B71 EPS, or PBS and transferred intravenously into BRPKp110-bearing mice on days 10, 14, and 18.

#### Germ-free assays

For germ-free colonisation experiments, mice were orally administered 1 × 10^10^ CFU of *B. pseudocatenulatum* 210 or PBS. Animals were sacrificed at 6, 24, and 48 h after administration and gut contents from the upper and lower colon, caecum, and small intestine were collected. A 50 mg aliquot of each sample was homogenised in 1 ml PBS and 100 μl of the resulting slurry was plated on MRS agar supplemented with cysteine and incubated anaerobically at 37°C for 48 h. Colonies were counted and bacterial load was calculated as CFU/g = (number of colonies × dilution factor)/(volume cultured × sample weight). PBS-treated germ-free controls were confirmed to remain sterile.

#### Bacterial culture

All *Bifidobacterium* strains were cultured at 37°C in MRS broth supplemented with L-cysteine (50 mg/L) under anaerobic conditions. Strains were grown for one week and preserved by lyophilisation during exponential growth. Individual dosage vials were stored at - 80°C, and viable counts were established by serial dilution and plating on MRS agar to determine the mean CFU per vial for *in vivo* dosing. For acid-killed preparations, bacterial suspensions were treated with 0.4% peracetic acid at room temperature for 1 h as described by Moor et al. ^34^, washed three times in sterile PBS, and resuspended to the required concentration. Loss of viability was confirmed by anaerobic culture.

#### BMDC culture

BMDCs were generated from bone marrow flushed from tibias and femurs of C57BL/6 mice (mixed from both male and female mice). Bone marrow suspensions were treated with red blood cell lysis buffer for 5 min, washed twice in PBS, and cultured overnight in RPMI-1640 medium supplemented with 10% FBS, 1% pen/strep, 20 ng/ml IL-4, and 10 ng/ml GM-CSF. On day 2, supernatants containing non-adherent cells were removed and replaced with fresh medium; medium was replaced again on day 5. Semi-adherent BMDCs were collected on day 7. For selected experiments, BMDCs were purified with CD11c MicroBeads according to the manufacturer’s instructions. For *in vitro* stimulation assays, BMDCs were incubated with the indicated concentrations of EPS, live bacteria, or PBS vehicle for the times indicated in the figure legends.

#### Histology

Harvested lungs were fixed overnight in 4% paraformaldehyde at 4°C, processed using a Leica tissue processor, embedded in paraffin, sectioned, and stained with hematoxylin and eosin according to standard histological procedures.

#### Lamina propria cells

Small intestine and colon were excised, opened longitudinally, cleared of luminal contents, and washed in PBS containing 2% FBS. Epithelial layers were removed by incubation in PBS containing 2% FBS, 0.1 mM DTT, and EDTA (2 mM for small intestine, 4 mM for colon) at 37°C with shaking for 10 min per round; two rounds were used for small intestine and three rounds for colon. Remaining lamina propria tissue was minced and digested in RPMI containing 2% FBS, 2 mM CaCl_2_, and collagenase D (0.25 mg/ml) for 10 min at 37°C with shaking. Digests were filtered through a 70 μm strainer, washed, and enriched over a 30%/100% isotonic Percoll gradient centrifuged at 670 × g for 30 min with low acceleration and no brake. Immune cells were collected from the interphase and washed before staining.

#### Flow cytometry

Tumours and lungs were mechanically dissociated. Tumours were digested in 0.2% collagenase IV and lungs in 0.2% collagenase I, each supplemented with 0.01% hyaluronidase and 0.01% DNase I, with digestion for 60 min (tumours) or 30 min (lungs) at 37°C under agitation. Spleens and lymph nodes were dissociated mechanically; blood was processed following collection into EDTA. Cells were washed, resuspended in FACS buffer (PBS supplemented with 1% FBS), and stained with extracellular antibodies listed in the Key Resources Table. For intracellular cytokine detection, cells were stimulated in RPMI 1640 medium containing 10% FBS, 50 μM 2-mercaptoethanol, 50ng/ml phorbol 12-myristate 13-acetate, 750ng/ml ionomycin, and 10μg/ml brefeldin A for 4 h before fixation and permeabilisation. Data were acquired on a BD LSR Fortessa and analysed in FlowJo. Gating strategies used to define the reported immune populations are described in Table 1.

#### Serum isolation

Blood was collected by cardiac puncture immediately after euthanasia. For serum preparation, blood was allowed to clot at room temperature and then centrifuged to separate serum, which was stored at − 80°C until downstream analyses.

#### Metabolomics

Untargeted serum metabolomics was performed by Biocrates (Innsbruck, Austria) using the MxP Quant 500 kit. Lipids and hexoses were measured by FIA-MS/MS and small molecules by LC-MS/MS on a SCIEX API 5500 QTRAP instrument with electrospray ionisation. Sample processing and quantitative analysis were performed according to the manufacturer’s workflow. Downstream analyses including principal coordinates analysis, heatmaps, and differential metabolite comparisons were carried out using MetaboAnalyst 4.0 and MetaboAnalyst 5.0 ^45, 46^.

#### MSD cytokines

For Mesoscale Discovery cytokine analysis, tumour tissues were homogenised in bead-beating tubes containing homogenisation buffer, clarified by centrifugation, and analysed using the indicated MSD multiplex assays (Key Resource Table) according to the manufacturer’s instructions.

#### Gut ELISAs

For intestinal cytokine and chemokine measurements, tissues were lysed in RIPA buffer containing protease inhibitors using a TissueLyser LT and Lysing Matrix D tubes. Lysates were clarified by centrifugation and total protein concentrations were determined using the DC Protein Assay. IL-12p70, CCL19, and CCL21 were quantified using DuoSet ELISA kits and Ancillary Reagent Kit 2 according to the manufacturer’s instructions. Plates were read at 450 nm with wavelength correction at 570 nm on a VersaMax microplate reader, and corrected absorbance values were calculated by subtracting 570 nm from 450 nm readings.

#### Tumour microbiology

For tumour microbiome analysis, murine mammary tumours were dissected aseptically, manually homogenised in PBS, and plated on Columbia blood agar, MRS agar, brain heart infusion (BHI) agar, and, for anaerobic culture, MRS agar with L-cysteine (50 mg/L), BHI agar with L-cysteine, and PYGS agar. Plates were incubated under aerobic or anaerobic conditions as appropriate. Environmental and skin-swab controls were included.

#### qPCR

Bacterial load of *B. pseudocatenulatum* 210 was estimated by qPCR using species-specific GroEL primers described by Junick and Blaut ^47^. Reactions were performed in duplicate using LightCycler 480 SYBR Green I Master with 10 μM primers and template DNA. Standard curves were generated using serial dilutions of monoculture-extracted DNA down to 0.001 ng/μl. Reactions were run on a LightCycler 480 and analysed using LightCycler 480 software.

#### EPS purification

EPS isolation and purification were based on the protocol of Ruas-Madiedo ^48^. Briefly, *Bifidobacterium* cultures were grown anaerobically on MRS-based agar, bacterial lawns were harvested in Milli-Q water, and biomass was extracted with NaOH. EPS was precipitated with ice-cold ethanol, redissolved, dialysed, and lyophilised to generate crude EPS. For purified EPS, nucleic acids and proteins were removed by sequential DNase I and Pronase E treatment, followed by trichloroacetic acid precipitation, dialysis, and lyophilisation.

#### AF647 EPS tracing

Purified 210 EPS was labelled with Alexa Fluor 647 hydrazide following mild periodate oxidation and hydrazide coupling. Concentrated EPS in sodium acetate buffer was oxidised with sodium periodate, quenched with ethylene glycol, and buffer exchanged using regenerated-cellulose centrifugal filter units. Alexa Fluor 647 hydrazide was added to oxidised EPS and reactions were incubated overnight at 4°C protected from light. Unbound fluorophore was removed by repeated washing. For tracing studies, age-matched female C57BL/6J mice were orally administered AF647-labelled 210 EPS (80 μg in 200 μl PBS), PBS alone, or free dye control. At the indicated time points, blood and organs were collected. Serum fluorescence was quantified against serially diluted AF647-EPS standards on a CLARIOstar Plus plate reader. *Ex vivo* fluorescence imaging of heart, lungs, liver, kidneys, spleen, and the full gastrointestinal tract was performed on an In-Vivo Xtreme multimodal optical and X-ray imaging system and images were processed in ImageJ.

#### RNA sequencing

BMDCs were seeded at 2.5 × 10^5^ cells per well in 24-well plates and stimulated for 6 h with purified 210 EPS (10 μg/well) or sterilised PBS. Total RNA was extracted using the RNeasy Micro Kit and RNA quality was assessed by NanoDrop. Library preparation and sequencing were performed by Genewiz/Azenta Life Sciences (Leipzig, Germany). Poly(A) + RNA was enriched using oligo-dT magnetic beads, cDNA libraries were prepared, size selected to approximately 370–420 bp, quality checked on an Agilent Bioanalyzer 2100, and sequenced on an Illumina NovaSeq platform to generate 150 bp paired-end reads. Raw FASTQ files were processed with FastX-toolkit, quality was assessed with FastQC, and adapter trimming and filtering were performed with Trimmomatic using a minimum Phred score of 20 ^49^. Reads were aligned to the Mus musculus GRCm38 genome using STAR v2.5.2b, gene counts were generated with featureCounts v1.5.0-p3 ^50^, and differential expression analysis was performed with DESeq2 v1.20.0 ^51^. GO and KEGG enrichment analyses were performed using clusterProfiler ^52^, and gene set enrichment analysis was run using the Broad Institute GSEA tool ^53, 54^.

#### EPS structural assays

Glycosyl composition and linkage analyses of purified 210 EPS and B71 EPS were performed by the Complex Carbohydrate Research Center (University of Georgia) by GC-MS using derivatised monosaccharide and partially methylated alditol acetate workflows, respectively. Size exclusion chromatography was performed in 50 mM ammonium acetate buffer on a Superose 6 Increase column with refractive index detection, and fractions were collected for downstream analysis. NMR spectroscopy of crude EPS and SEC fractions was carried out following repeated exchange into D_2_O. One-dimensional and two-dimensional spectra were acquired to define principal structural motifs.

#### On-cell STD NMR

For on-cell Saturation Transfer Difference (STD) NMR, non-adherent BMDCs were prepared as described above. Briefly, 5 × 10^6 cells were pelleted (300 × g). EPS210 fractions were prepared in 450 μL RPMI medium supplemented with 50 μL D2O (98.9%, Merck) and added to the cell pellets to a final EPS concentration of 0.4 mM. Samples (500 μL) were transferred to sterile NMR tubes (Wilmad 528-PP-7 series) and analysed on a Bruker Avance NEO spectrometer operating at a ^1^H frequency of 500.11 MHz. Spectra were acquired at 310 K with continuous spinning to maintain cells in suspension.

On-Cell STD experiments were performed using the Bruker’s *stddiffesgp.3* pulse sequence, featuring 2.5 and 5 ms trim pulses, 3 ms spoil gradient and water suppression using gradient excitation sculpting. Saturation was done using a train of 50-ms 0.4 mW Gaussian pulses in the on-resonance frequency channel at 0 ppm and 40 ppm in the off-resonance. The broad protein peaks were removed using a 40 ms spinlock filter. STD build-up curves were performed using saturation times 0.5-6 s with recycle delay of 7 s. All experiments were performed with 4 dummy scans and 128 scans. The STD (%) factor for each EPS fraction was determined using the ^1^H peaks in the 3.8–3.4 ppm range, after subtracting the RPMI media background.

#### Whole-genome sequencing

Bacterial genomic DNA was extracted using the FastDNA SPIN Kit for Soil. Whole-genome sequencing of *B. pseudocatenulatum* LH14 and *B. longum subsp. longum* B71 was performed at the Wellcome Trust Sanger Institute as previously described by Lawson et al. ^40^ and Kujawska et al. ^39^. *B. pseudocatenulatum* 210, *B. bifidum* LH80, *B. choerinum* LH506, and *B. longum subsp. longum* NCIMB 8809 were sequenced at the Quadram Institute Bioscience on the Illumina NextSeq500 platform; NCIMB 8809 was additionally sequenced using Oxford Nanopore technology.

#### Comparative genomics

Sequencing reads for *B. pseudocatenulatum* 210, *B. bifidum* LH80, and *B. choerinum* LH506 were pre-processed with fastp v0.22 ^42^, assembled with Unicycler v0.5.0 ^55^, and assessed with CheckM v1.2.3 ^56^. Genomes were annotated with Prokka v1.14.6 ^57^. BLAST+ v2.13.0 ^58^ was used to screen genomes for EPS-associated loci using previously described eps gene sets ^59^ and additional reference loci. Gene-cluster similarity visualisation was performed with clinker and clustermap.js ^60^.

### QUANTIFICATION AND STATISTICAL ANALYSIS

#### Statistics

Statistical analyses were performed using GraphPad Prism 11 unless otherwise stated. Normality was assessed using Kolmogorov-Smirnov testing where appropriate. Two-tailed unpaired t tests, Mann-Whitney tests, one-way ANOVA with post hoc multiple-comparisons testing, and Kaplan-Meier analysis were applied as described in the figure legends. Sample size (n), what n represents, and exact statistical tests are indicated in the corresponding figure legends. Differences were considered statistically significant at P < 0.05 unless otherwise indicated.

## Figures and Tables

**Table 1:** List of flow cytometry gating strategies for the identification of immune cell populations

Population	Gating strategy
Lymphoid cells	Singlet, Live, CD45+, CD3+
T helper cells	Singlet, Live, CD45+, CD3+, CD4+, CD8−
CD8^+^ T cells	Singlet, Live, CD45+, CD3+, CD4−, CD8+
CD8^+^ effector memory	Singlet, Live, CD45+, CD3+, CD4−, CD8+, CD62L−, CD44+
CD8^+^ central memory	Singlet, Live, CD45+, CD3+, CD4−, CD8+, CD62L+, CD44+
Naïve CD8	Singlet, Live, CD45+, CD3+, CD4−, CD8+, CD62L+, CD44−
Treg cells	Singlet, Live, CD45+, CD3+, CD4+, CD8−, FOXP3+
NK cells	Singlet, Live, CD45+, CD−, NK1.1+
Myeloid cells	Singlet, Live, CD45+, CD11b+
M-MDSCs	Singlet, Live, CD45+, CD11b+, Ly6C+, Ly6G−
G-MDSCs	Singlet, Live, CD45+, CD11b+, Ly6C−, Ly6G+
Macrophages	Singlet, Live, CD45+, CD11b+, Ly6C−, F4/80+
Dendritic cells (DCs)	Singlet, Live, CD45+, CD11c+
cDC1 cells	Singlet, Live, CD45+, CD11c+, MHCII+, CD103^hi^, CD11b^lo^
cDC2 cells	Singlet, Live, CD45+, CD11c+, MHCII+, CD103^lo^, CD11b^hi^
Gut cDC1 cells	Singlet, Live, CD45+, CD11c+, MHCII+, CD103^hi^, CD11b^lo^, XCR1^+^
Gut cDC2 cells	Singlet, Live, CD45+, CD11c+, MHCII+, CD103^lo^, CD11b^hi^, SIRPα^+^

## Data Availability

This study did not report custom code. Software packages used for sequencing, metagenomic, transcriptomic, structural, imaging, and statistical analyses are listed in the Key Resources Table and described below. Requests for additional information required to reanalyse the datasets reported in this study should be directed to the lead contacts.
